# Calcium Hydroxyapatite as a Co-adjuvant Treatment Option in a Patient With Morphea: A Report of a Case With a One-Year Follow-Up

**DOI:** 10.7759/cureus.69741

**Published:** 2024-09-19

**Authors:** Carolina Schneider, Luis A Parra Hernandez, Eugenia Cure, Ingrid Salas, Andrea M Parra

**Affiliations:** 1 Plastic Surgery, Dra Carolina Schneider Plastic Surgery, Buenos Aires, ARG; 2 Aesthetic Medicine, Sociedad Internacional De Rejuvenecimiento Facial No Quirúrgico (SIRF), Barranquilla, COL; 3 Aesthetic Medicine and Alternative Medicine, Dra Eugenia Cure Aesthetic Medicine, Barranquilla, COL; 4 Dermatologist, Dermatosphera Skinclinic, Cartagena, COL; 5 Oculoplastic Surgery and Aesthetic Medicine, Sociedad Internacional De Rejuvenecimiento Facial No Quirúrgico (SIRF), Barranquilla, COL

**Keywords:** calcium hydroxyapatite, clinical case report, filler injection, localized scleroderma, morphea

## Abstract

Morphea, or localized scleroderma, is a chronic inflammatory condition that unequivocally affects the dermis and subcutaneous connective tissue. It undeniably causes significant disfigurement in approximately half of patients, profoundly impacting their self-esteem. The available treatment options include corticosteroids (taken orally or administered subcutaneously), phototherapy, CO_2_ fractional laser treatment, and biologically mediated medications. It is crucial to note that using fillers as adjuvant therapy for inflammatory diseases indisputably raises concerns due to the potential to trigger inflammation and lead to disease reactivation.

In one case, a 24-year-old patient with morphea on her face underwent a combined approach involving plastic surgery, dermatology, and regenerative aesthetics treatment with lipo-filling initially by an expert plastic surgeon. Then, after reviewing the literature and consensus from the dermatologist, aesthetics physician, and alternative medicine expert, it was decided to use calcium hydroxylapatite-carboxymethylcellulose (Radiesse, Merz Pharmaceuticals GmbH, Frankfurt, Germany) in the affected area. After a year of follow-up, there was a significant improvement in the appearance of her face and skin, as confirmed by a 10-point improvement on an activity measuring scale.

Additional research will solidify whether calcium hydroxylapatite (CaHA) is the optimal injectable for treating dermal autoimmune diseases. Our initial approach demonstrates significant promise for regenerative biostimulation. Through collaboration, we have effectively integrated plastic surgery techniques, fillers, dermatologists, and alternative medicine perspectives to treat inflammatory diseases, providing a comprehensive and robust exploration of morphea treatment.

## Introduction

Localized scleroderma, or morphea, is characterized by thickened and hardened skin patches and has a self-limiting course [1]. The thickened skin can usually be observed in spots and bands on the face, scalp, trunk, legs, or arms. Morphea is categorized as a skin-limited condition; however, it has the potential, based on the subtype and affected body part, to lead to noticeable disfigurements such as hyperpigmentation and skin atrophy [1,2]. Around half of the patients experience disfigurement, and this can have a significant adverse effect, potentially leading to decreased self-confidence [3] as well as physical limitations like joint contractures [2].

The prevalence of morphea ranges from 0.4 to 2.7 cases per 100,000 in adults and 0.3 to 3 cases per 100,000 in children. It is more common in women, occurring 2.6 to 6 times more frequently in women than men, and usually appears in adults between the ages of 40 and 50. The usual course of the condition starts with skin redness during the initial stage of inflammation. Then, it progresses to fibrosis, sclerosis, and atrophy, accompanied by changes in skin pigmentation to a lighter or darker shade [4].

The causes of scleroderma are diverse and may involve environmental factors, trauma, and genetic predisposition, which can lead to immune and fibrotic pathway dysregulation. It is important to note that this condition does not typically progress to systemic sclerosis, although co-occurrence of the two conditions has been reported [4]. There have also been reports of familial clustering and the coexistence of localized scleroderma with autoimmune diseases, such as Hashimoto's thyroiditis, alopecia areata, vitiligo, type 1 diabetes, and genital lichen sclerosis, suggesting a potential genetic component [5].

Therapeutic options for treating the condition involve oral or subcutaneous methotrexate, corticosteroids, calcipotriol, imiquimod, tacrolimus, CO_2_ fractional laser treatment, mycophenolate mofetil, and medium-dose UVA1 phototherapy [6]. Additionally, more recent medications target cytokines, immune-related receptors, the adaptive immune system, cellular and gene therapy, and even agents targeting cellular senescence, aging, and death analytics. These genetic and biological therapies are still under study and are based on a thorough understanding of the disease's causes, but they are yet to be available to all patients [2]. Some authors have reported their experience treating disease-associated facial deformities with hyaluronic acid (HA) and polylactic acid-based injectables [7,8]. Using dermal fillers in connective tissue disease (CTD) is still debated due to the theoretical risk of antigenic stimulation causing disease reactivation or worsening [9].

Calcium hydroxylapatite with carboxymethylcellulose (CaHA-CMC; Radiesse, Merz Aesthetics, Raleigh, NC, USA) has numerous benefits, such as cell proliferation, elastic fiber and elastin expression, angiogenesis, collagen production, improvement of skin elasticity, mechanical proprieties, and wound healing processes [10]. It is known worldwide as a dual-purpose filler with regenerative and biostimulation capabilities [11]. This extends beyond simply adjusting volume; instead, enhancing the skin's flexibility by increasing the generation of extracellular matrix (ECM) elements such as collagen 1 and 3, elastin, proteoglycans, and fresh blood vessels [12,13]. CaHA-CMC, adeptly suited for soft tissue regeneration, promotes the activation of endogenous fibroblasts, whether in its undiluted, diluted, or hyperdiluted form.

Research studies indicate that patients with connective tissue diseases tolerate CaHA products well. Cox SE et al. treated a 16-year-old boy with Parry-Romberg syndrome using CaHA due to its substantial volume restorative properties. They administered four 3.9 mL injections and one 2.6 mL injection of CaHA, approximately four weeks between sessions, followed by a 1.0 mL injection of HA. This process resulted in significant recolonization, and the patient showed continued clinical improvement at the six-month follow-up [11].

However, there is currently no available literature on the safety and efficacy of CaHA filler in morphea. This study presents a collaboration of authors from diverse affiliations: plastic and oculoplastic surgery, dermatology, alternative medicine, and aesthetics medicine. This reflects the interdisciplinary nature of treatment approaches and the benefits of combining expertise from various fields and institutions. By bringing together experts, we aim to comprehensively understand morphea co-adjuvant treatments with regenerative aesthetic medicine that leverages each co-author's unique perspectives and strengths.

Ultimately, our collaborative effort demonstrates the value of interdisciplinary option evaluations in advancing our understanding of morphea treatment options, particularly emphasizing skin quality and aesthetic results.

## Case presentation

**Figure 1:**
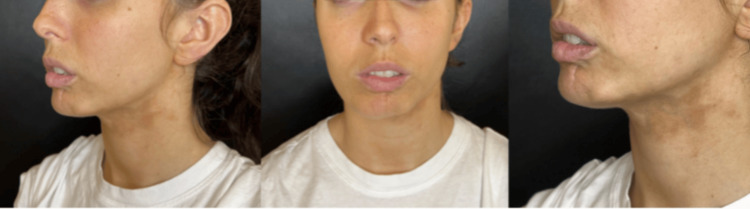
Patient's first photo. Before photo of a 24-year-old patient with morphea on the left side of the face, creating skin contraction on her lower lip and the Jow line.

We present the case of a patient treated in Buenos Aires, Argentina, in the clinic of Dr María Carolina Schneider - plastic and aesthetic surgery. A 24-year-old female patient came in March 2023 with a morphea plaque on her face, at her left facial contour, extending to the submental region; this plaque caused retraction, especially on her lower lip left side, discomfort and aesthetic changes that the patient wants to improve, according to previous dermatological evaluation she has Localized Scleroderma Cutaneous Assessment Tool (LoSCAT) of 15/18. She previously received complete treatment with oral corticoids, tacrolimus, and UVA phototherapy every six months for three years without aesthetic results. After obtaining a complete photographic record and written consent to use her image and data in future retrospective scientific research (Figure 1)

**Figure 2:**
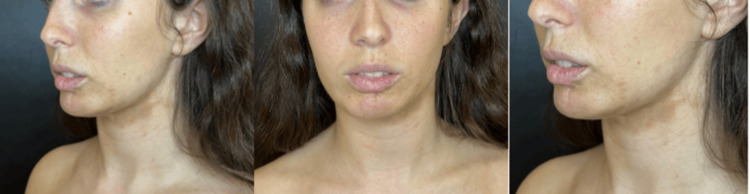
Post-surgery photo. Three months after facial Lipofilling surgery in August 2023, the patient presents skin retraction associated with morphea

Initial analysis indicated that she was an ideal patient for a surgical procedure: facial lipofilling was performed in May 2023 by extracting 100 ccs of abdominal fat with a 2 mm cannula, then a total of 20 ccs of fat was placed in the chin, jawline and lower hemi labium using 1.2 mm cannula. The procedure was well-tolerated and did not present any complications; however, the traction, especially on the lower lip on her left side, and the changes and skin colorations secondary to the dermal lesion of morphea persisted (Figure 2). 

**Figure 3:**
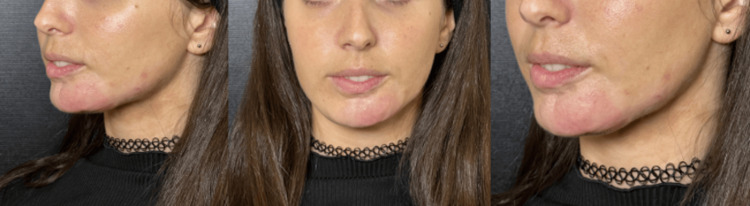
Post- CaHA Photo. December 2023, one month after biostimulation with one hydroxyapatite CaHA-CMC (Radiesse, Merz Pharmaceuticals GmbH, Frankfurt). The improved skin quality, dermal thickness, and coloring in the treated area can be seen. CaHA-CMC: calcium hydroxylapatite-carboxymethylcellulose

In November 2023, calcium hydroxylapatite-carboxymethylcellulose (CaHA-CMC; Radiesse from Merz Pharmaceuticals GmbH, Frankfurt, Germany), was applied to the area using a standard dilution: 1.5 ml CaHA + 1.5 diluent. The product was applied with a 22 G cannula at the subdermal level, leaving microinjections with a small amount of product. The patient tolerated the procedure well, and there were no associated complications. One month later, the photographic record shows an outstanding response with improvement in the coloring of the morphea plaque, increased skin laxity, a significant increase in dermal thickness, and improvement in the retraction of the lower lip, also presented redness in the area, as CaHA typically leads to edema and angiogenesis favors erythema (Figure 3).

The result was so positive that it was decided to apply another syringe of CaHA-CMC using the same dilution.

**Figure 4:**
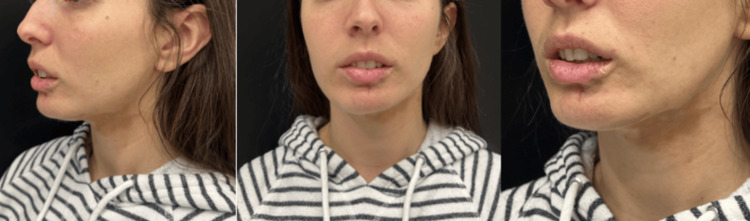
Follow-up of CaHA treatment Follow-up in May 2024, six months after the first treatment with Radiesse, with improvement in skin retraction and coloration. CaHA: calcium hydroxylapatite

After this improvement and due to the excellent clinical response, it was decided to carry out a three-month follow-up. Upon evidence that the disease was not reactivated or that there was no undesired reaction, a second application of CaHA-CMC in a 1:1 dilution was carried out in February 2024 (Figure 4).

Results

**Figure 5:**
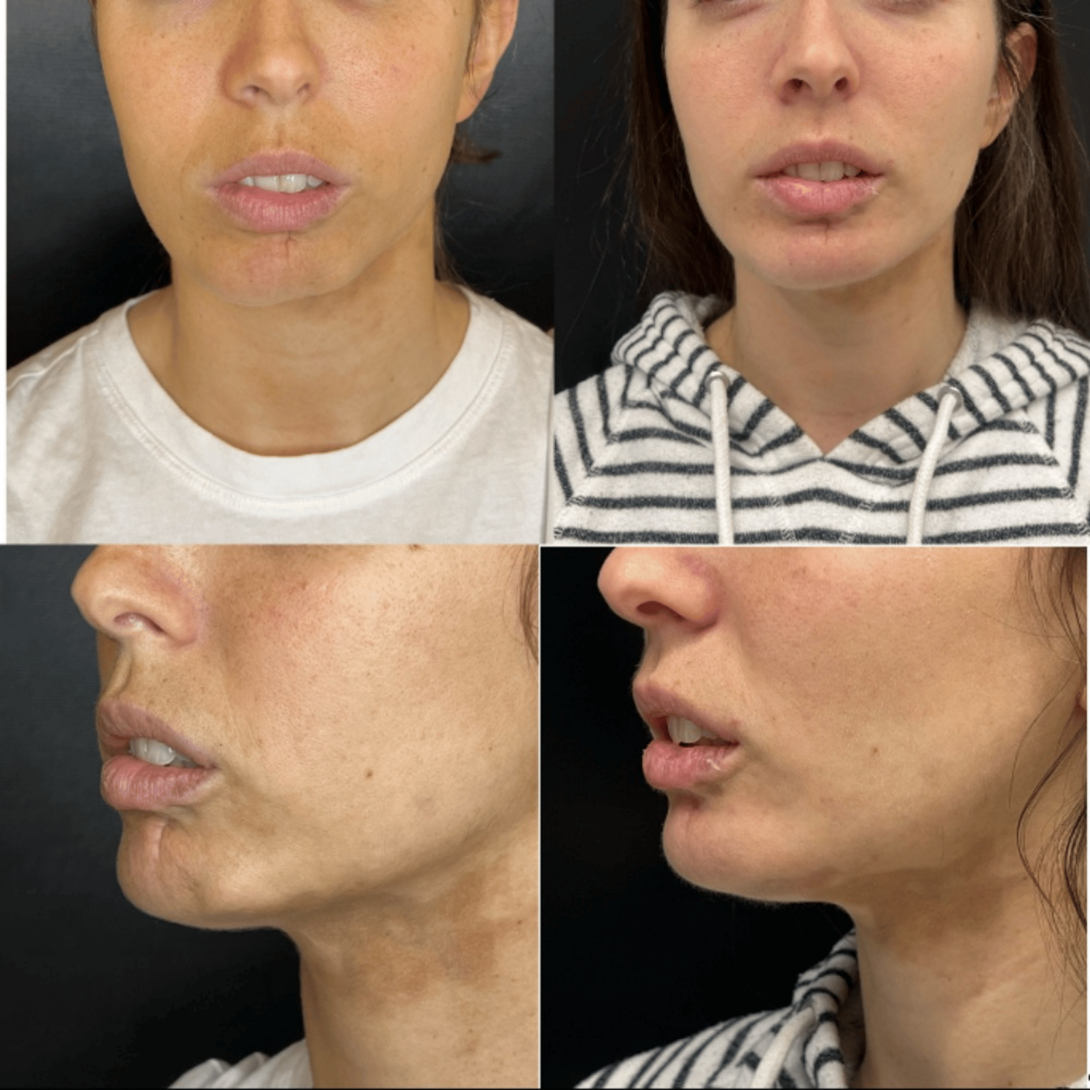
Final follow-up A: Before photo in May 2023 B; after photo in May 2024, the one-year follow-up, six months from the first CaHA treatment and three months from the second CaHA treatment. There is an improvement in mandibular contour, a decrease in lower lip retraction, and a marked improvement in skin colouration. CaHA: calcium hydroxylapatite

After complete oral and dermal treatment for more than three years without a response, in 2023, this patient was taken to lipofilling surgical management but persisted with skin changes. She received two sessions of CaHA-CMC from Merz Pharmaceuticals GmbH: one in November 2023 and one in February 2024. In both sessions, 1.5 ml of CaHA was diluted with 1.5 ml of standard saline solution and lidocaine without epinephrine and applied using a 22G cannula specifically in the area affected by morphea. After these two applications, there was a noticeable improvement in the skin in the affected area, along with a reduction in the retraction of the lower lip and a more symmetrical appearance of the lower third of the face overall (Figure 5). 

The authors used the Localized Scleroderma Cutaneous Assessment Tool (LoSCAT) to objectively measure the patient's condition [12]. This tool has been validated to assess disease activity by measuring the Localized Scleroderma Damage Index (LoSDI) and the Localized Scleroderma Severity Index (LoSSI) [13]. It evaluates six items: new lesion or growth, thickening, erythema, dermal atrophy, subcutaneous atrophy, and depigmentation, with a maximum score of 18. A higher score indicates an increase in disease activity [13]. For our patient, before LoSCAT, it was 15/18; after the treatment, it was only 5/18, an improvement of 10 points. There were no adverse reactions or complications.

## Discussion

Morphea is a persistent inflammatory condition that affects the dermis and subcutaneous connective tissue. It leads to a sclerotic process, causing widespread thickening and hardening of the skin and atrophy at different depths [12]. In this case report, a collaboration of authors from diverse affiliations: plastic and oculoplastic surgery, dermatology, alternative medicine, and aesthetics medicine share the experience of using CaHA-CMC from Merz Pharmaceuticals GmbH. Two separate sessions over a year on a 24-year-old female patient improved skin morphea plaque, with significant clinical and aesthetic improvement with no associated complications or reactivation of their underlying disease. Reflecting the interdisciplinary nature of treatment approaches and the benefits of combining expertise from various fields and institutions.

As an autoimmune disease, using fillers to address its dermis symptoms raises concerns about triggering inflammation and disease reactivation. According to global literature, no absolute contraindication exists to use fillers in cases of scleroderma [4,14]. However, there is not enough evidence to support their safety, so physicians who are interested in these options must be prepared to choose the perfect filler for the injection in morphea patients. Generally, it should be non-immunogenic, biocompatible, and stable at the implantation site, with minimal risk of complications while treating the ECM.

Calcium hydroxylapatite with carboxymethylcellulose is a dual-purpose filler with regenerative and biostimulation capabilities [15]. This blend of treatments extends beyond simply adjusting volume, instead enhancing the skin's flexibility by increasing the generation of ECM elements such as collagen 1 and 3, elastin, proteoglycans, and fresh blood vessels [16,17]. CaHA-CMC, adeptly suited for soft tissue regeneration, promotes the activation of endogenous fibroblasts, whether in its undiluted, diluted, or hyperdiluted form [18].

According to publications, CaHA-CMC has been used before in autoimmune diseases, like Parry-Romberg syndrome, with maintained clinical improvement at the six-month follow-up [11]. Even though it is a different illness, it presents the opportunity to explore the use of biostimulators in this disorder. The authors initially conducted a comprehensive analysis of the cellular mechanism of the disease to consider the use of CaHA in this patient. Wenzel et al.'s findings discovered that an unidentified stimulus causes tissue damage in morphea, leading to the secretion of chemokines that attract leukocytes to the dermis. This process results in the release of cytokines such as transforming growth factor beta (TGFβ), interleukin 1 (IL‐1), and IL‐6, as well as IL‐4, 8, and 13, which are crucial for the differentiation of precursor cells, including fibroblasts, pericytes, and endothelial cells, into myofibroblasts [19]. The excessively activated myofibroblasts produce a surplus of collagen, fibronectin, and tenascin‐C. These substances act as damage-associated molecular patterns (DAMPs) and have the potential to activate undifferentiated fibroblasts, causing them to transform into myofibroblasts. This self‐fibrotic process enables the unregulated proliferation of fibroblasts [2].

## Conclusions

Morphea, or localized scleroderma, is a skin condition characterized by the thickening and hardening of skin areas due to excessive collagen deposition. This can have significant aesthetic impacts, especially on the face. Surgical interventions like lipofilling have been explored, but aesthetic improvement is sometimes lacking even after complete treatment. Recently, calcium hydroxylapatite fillers have shown regenerative properties beyond collagen, producing elastin and proteoglycans and promoting angiogenesis and wound healing. This makes CaHA-CMC fillers a promising adjunct therapy for improving the appearance of facial morphea. However, as morphea is an autoimmune disease, the safety and effectiveness of this approach have not been wholly described in clinical publications. Physicians must carefully weigh the risks and benefits when considering CaHA filler treatment for patients with morphea.

This case report described the authors' experience using CaHA-CMC filler as an adjunct therapy for a 24-year-old patient who had previously received oral and topical treatments for two years and a surgical lipofilling intervention in May 2023 but did not achieve satisfactory aesthetic outcomes. After applying the first CaHA-CMC filler in November 2023, the patient presented with redness that may have resulted from angiogenesis and initial swelling but also released the tension on her lower lip, which was not seen before with the other treatments she received. Three months later, a second CaHA-CMC treatment was performed, and the asymmetry continued to improve. The patient did not require additional treatments throughout the one-year follow-up and maintained satisfactory results. While this is just a single case, it suggests that calcium hydroxylapatite fillers can be a safe and effective option for improving the appearance of facial morphea, working as a coadjuvant option due to their capacity to stimulate fibroblast activity and new collagen production while enhancing angiogenesis, the critical impact that it had on elastic fibers that may facilitate to improved skin contractures, and the regenerative actions that impact the extracellular matrix al these together a multidisciplinary approach with plastic surgery and dermatological treatment present a promising option. Further research with larger sample sizes is needed to evaluate the safety and efficacy of this approach entirely. 
